# Mechanical Thrombectomy in Pediatric Large Vessel Occlusions Before Cerebrovascular Maturity: A Case Report and Technical Note

**DOI:** 10.7759/cureus.59027

**Published:** 2024-04-25

**Authors:** Nicholas P Derrico, Rebekah Kimball, Kristin Weaver, Allison Strickland

**Affiliations:** 1 Neurosurgery, University of Mississippi Medical Center, Jackson, USA

**Keywords:** pediatric congenital heart disease, mechanical thrombectomy in pediatrics, basilar occlusion, large vessel occlusion (lvo), pediatric stroke

## Abstract

Pediatric arterial ischemic stroke is a rare but increasingly acknowledged disorder. Large vessel occlusions in this population have been treated off-label with endovascular thrombectomy. However, there is limited evidence to guide management. Small children, before the age of five when the cerebrovasculature reaches adult size, present additional challenges.

We report the case of cardioembolic basilar occlusion in a two-year-old and the technical details of endovascular management, currently lacking in published literature. We employed a 5 French slender sheath, typically used for radial access, as a femoral short sheath. We accessed the dominant vertebral artery with a 5 French intermediate catheter, navigated with a typical 0.027-inch microcatheter and 0.014-inch microwire, and performed direct aspiration thrombectomy of the basilar clot. No closure device was employed. The patient had a near-complete and durable recovery.

Small children present additional challenges for the endovascular management of stroke. Pre-procedural imaging can be used to design an aspiration-capable system appropriate for the child’s size. Endovascular thrombectomy in children is feasible with some modifications to adult protocols.

## Introduction

Arterial ischemic stroke is a rare but increasingly acknowledged disorder in pediatric patients. While the majority of pediatric strokes affect the neonatal population, strokes in childhood have an incidence of 2-8/100,000 children per year [1,2]. The underlying etiology for these strokes is usually cardioembolic causes when compared to the adult population. Up to 25% of childhood strokes have underlying congenital heart disease [3]. Other risk factors include inflammatory vasculitides, prothrombotic disorders, and head and neck trauma [4]. 

Large vessel occlusions (LVOs) in children have been treated with endovascular thrombectomy with good results [5]. Thrombectomy is an off-label intervention that is not Food and Drug Administration-approved (FDA) for use in childhood stroke [6]. However, the American Heart Association/American Stroke Association has noted that it is reasonable to consider intervention in larger children with persistent disabling neurologic deficits and radiographically confirmed cerebral LVO [7]. The lower limit of *larger children* has not been defined, though cerebral vasculature reaches an adult size on angiography by approximately five years old [8]. There is limited evidence to guide management in pediatric strokes with LVO before this age as the largest case series to date for pediatric thrombectomy only includes 13 cases under the age of five [5]. Interventions in smaller children require additional considerations, and the best techniques to perform thrombectomy in this cohort have not been elucidated.

Pediatric stroke disproportionately affects the posterior circulation, accounting for up to half of all pediatric strokes [9]. Thrombectomy in the posterior circulation is also not FDA-approved [10]. The best management of these lesions is actively studied in the adult literature with some evidence that direct aspiration has improved recanalization rates with a trend toward less intracerebral hemorrhage relative to stent retrievers [11].

This case report aims to explore the management of pediatric endovascular stroke intervention in patients before cerebrovascular maturity. We present the case of a direct aspiration thrombectomy in a two-year-old girl with posterior circulation LVO, including a technical discussion.

## Case presentation

**Figure 1:**
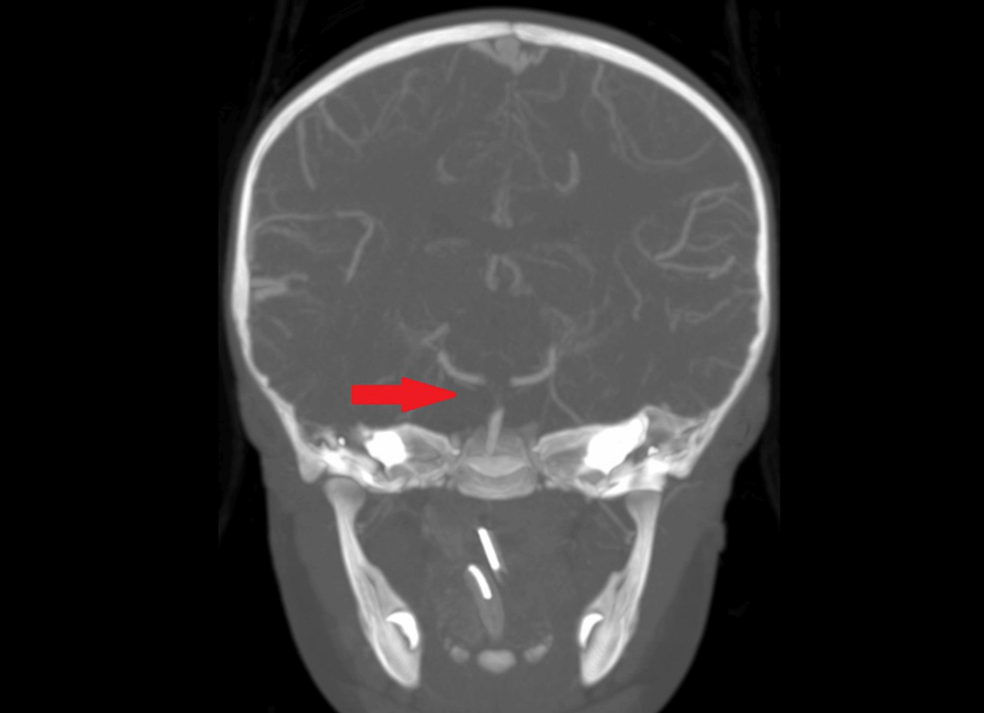
Coronal maximum intensity projection computed tomography angiography demonstrating distal basilar occlusion.

A two-year-old girl with a history of hypoplastic left heart syndrome and Glenn bidirectional shunt was admitted to the pediatric cardiology service from the clinic when a large left ventricular thrombus was identified on a routine echocardiogram. In addition to her home dose of Aspirin 81 mg daily, she was initiated on a heparin infusion, with the plan to transition to therapeutic low-molecular-weight heparin. However, on hospital day three she had an exam change and was found unresponsive. She was bolused 75 units/kg of heparin, intubated, and urgently evaluated. A non-contrast CT head was normal, and a CT angiogram demonstrated a distal basilar occlusion involving both pre-communicating posterior cerebral arteries (PCAs) (Figure 1). The posterior communicating arteries were patent bilaterally, as were the distal PCAs. There was a faint filling of the superior cerebellar arteries (SCAs) bilaterally. There was also a focal occlusion of the nondominant right distal foraminal segment of the vertebral artery with retrograde filling. The initial neurosurgical evaluation was limited by neuromuscular paralysis from recent intubation, but she was anisocoric with pupils 5 mm and nonreactive on the right and 3 mm and sluggish on the left. Consent was obtained from the mother at the bedside, and she was taken emergently for a thrombectomy. Thrombolytics were not given as she was already anticoagulated.

**Figure 2:**
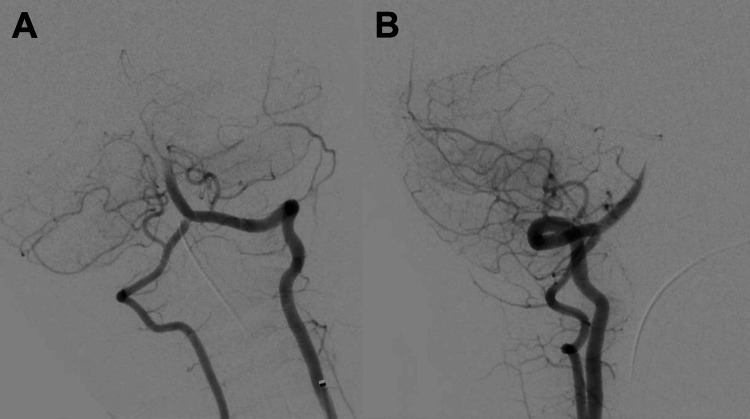
Initial diagnostic selective angiogram of the left vertebral artery demonstrating distal basilar occlusion: (A) anteroposterior and (B) lateral projections. There is no filling of the posterior cerebral arteries, but there is trace flow through the superior cerebellar arteries. The right vertebral artery clot has recanalized.

Access was obtained via the right common femoral artery with a 5 French (Fr) Glidesheath Slender (Terumo). The dominant left vertebral artery was selected and a 5 Fr Sofia distal access catheter (Microvention) was ascended over a Glidewire into the distal foraminal segment of the vertebral artery. Of note, no guide catheter was used to limit the system size. The Sofia catheter was selected due to its 17 cm tip that was pliable enough to navigate the third and fourth segments of the vertebral artery. Diagnostic runs demonstrated a complete distal basilar occlusion similar to the CT angiogram, with a very sluggish anterograde filling of the SCAs. The focal occlusion of the right vertebral artery had resolved (Figure 2).

**Figure 3:**
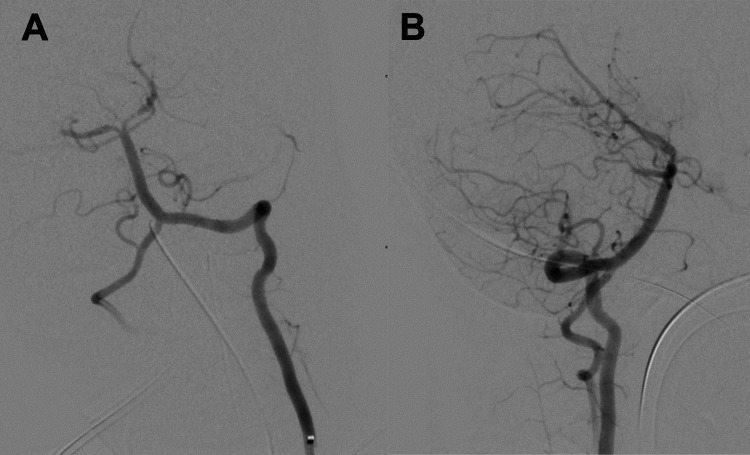
Follow-up selective angiogram of the left vertebral artery after aspiration of distal basilar occlusion: (A) anteroposterior and (B) lateral projections. The posterior cerebral and superior cerebellar arteries fill with vasospasm in the left posterior cerebral artery, and thrombolysis in cerebral infarction grade 2B recanalization.

A Marksman 0.027-inch microcatheter (Medtronic) and Synchro soft 0.014 wire (Stryker) were used to navigate intradurally, guiding ascent of the Sofia aspiration catheter to the mid-basilar. Constant aspiration was initiated with the Penumbra Max system, and the catheter was withdrawn from the body. A visible clot was identified. Follow-up angiography demonstrated vasospasm in the left PCA but the near-complete filling of the basilar apex, pre-communicating and distal PCAs, and SCAs (Figure 3). The catheter system was withdrawn and manual pressure was held on the artery without a closure device. A post-procedural examination demonstrated eye-opening to voice, equal and reactive pupils, localizing antigravity in bilateral upper extremities, and spontaneous movement in bilateral lower extremities.

Magnetic resonance imaging (MRI) demonstrated scattered bilateral posterior circulation infarcts without any complete vascular territory compromise. Heparin was temporarily held after the procedure and restarted the next day. She was extubated on postoperative day 3. The left ventricular thrombus had largely resolved with some residual on the aortic root. She was ultimately discharged on therapeutic low-molecular-weight heparin injections and Aspirin 81 mg daily. A hematologic workup for thrombophilias was unremarkable, and she was discharged home.

At both one- and six-month follow-ups, she had a durable near-complete neurologic recovery. Her only residual neurologic deficit was a partial right third nerve palsy leading to exotropia that required patching. The cardiac thrombus persisted until her six-month follow-up when it was no longer detected. She underwent two additional brief hospitalizations: one to transition from low-molecular-weight heparin to rivaroxaban, and the other for acute otitis media during the interim period. No further episodes or hospitalizations were suspicious for cerebrovascular disease.

## Discussion

Acute ischemic stroke in childhood is a rare and potentially life-threatening condition. The best management of these strokes is not well-elucidated in the literature and remains at the discretion of the treating physician. While the efficacy of thrombectomy in pediatric patients has been demonstrated retrospectively, the constituent studies predominantly represent adolescent and teenage patients [5]. In children under five years old, whose vessels have not yet approached their adult size, special considerations must be made. In particular, special attention should be given to optimizing catheter systems to vessel size. In this case, that included using a smaller aspiration catheter than typical, forgoing a guide catheter, and using a slender sheath.

In this case, we treated a posterior circulation LVO in a child before cerebrovascular maturity with a small system typically used for radial access and an aspiration-only approach. On evaluating the preprocedural imaging, we determined that an attempt at thrombectomy was feasible given the left vertebral artery provided access to the basilar tip and was >3 mm in size, large enough to accommodate a 5 Fr aspiration catheter. We elected for femoral access with a 5 Fr Glidesheath slender. While often used for radial access, this sheath system has a thin and flexible wall to both limit the arteriotomy and allow easy tracking into the vessels of the pelvis. An aspiration-only technique was chosen for two reasons. First, our experience with adults suggests that stent-retrievers carry an increased risk of complication for thrombectomy in the posterior circulation, as has been previously reported [11]. Second, at angiography, the right vertebral clot had resolved. This was thought to be due to the timely administration of the heparin bolus before neurosurgical involvement. Suspecting a soft cardioembolic clot that was already starting to resolve, aspiration was deemed most appropriate.

## Conclusions

Despite the rare occurrence of LVOs in children, endovascular thrombectomy is feasible with some modifications to the typical thrombectomy protocols used in adults.In particular, the use of catheters specifically sized for the target vessel on preprocedural imaging and exploiting opportunities to downsize the system are crucial.

## References

[1] M Giroud, M Lemesle, J B Gouyon, J L Nivelon, C Milan, R Dumas (1995). Cerebrovascular Disease in children under 16 years of age in the city of Dijon, France: a study of incidence andclinical features from 1985 to 1993. J Clin Epidemiol.

[2] Kittner SJ, Adams RJ (1996). Stroke in children and young adults. Curr Opin Neurol.

[3] DeVeber G (2003). Risk factors for childhood stroke: little folks have different strokes!. Ann Neurol.

[4] Bernard TJ, Goldenberg NA (2010). Pediatric arterial ischemic stroke. Hematol Oncol Clin North Am.

[5] Bhatia K, Kortman H, Blair C (2019). Mechanical thrombectomy in pediatric stroke: systematic review, individual patient data meta-analysis, and case series. J Neurosurg Pediatr.

[6] Amlie-Lefond C, Wainwright MS (2019). Organizing for acute arterial ischemic stroke in children. Stroke.

[7] Ferriero DM, Fullerton HJ, Bernard TJ (2019). Management of stroke in neonates and children: a scientific statement from the American Heart Association/American Stroke Association. Stroke.

[8] He L, Ladner TR, Pruthi S, Day MA, Desai AA, Jordan LC, Froehler MT (2016). Rule of 5: angiographic diameters of cervicocerebral arteries in children and compatibility with adult neurointerventional devices. J Neurointerv Surg.

[9] McCrea N, Saunders D, Bagkeris E, Chitre M, Ganesan V (2016). Diagnosis of vertebral artery dissection in childhood posterior circulation arterial ischaemic stroke. Dev Med Child Neurol.

[10] Barry M, Hallam DK, Bernard TJ, Amlie-Lefond C (2019). What is the role of mechanical thrombectomy in childhood stroke?. Pediatr Neurol.

[11] Xenos D, Texakalidis P, Karras CL, Murthy NK, Kontzialis M, Rivet DJ, Reavey-Cantwell J (2022). First-line stent retriever versus direct aspiration for acute basilar artery occlusions: a systematic review and meta-analysis. World Neurosurg.

